# Synthesis of NiCo_2_O_4_ supported on Chitosan for potential adsorption of copper ions in water samples

**DOI:** 10.1038/s41598-025-96777-y

**Published:** 2025-04-24

**Authors:** Nadia E. A. El-Gamel, Shymaa S. Medany, Mahmoud A. Hefnawy

**Affiliations:** Chemistry Department, Faculty of Science, Cairo University, Giza, 12613 Egypt

**Keywords:** Copper adsorption, Chitosan, Spinel oxide, Nano-Adsorbent, Chemistry, Environmental chemistry

## Abstract

The present work involved the synthesis of nano-spinel oxide, which was subsequently incorporated into chitosan to enhance the adsorption efficiency of Cu(II) from aqueous samples. The chitosan was compared with the modified chitosan (nickel cobaltite@Chitosan) in terms of adsorption performance toward Cu (II) ions. Structural and morphological characterization was investigated using X-ray powder diffraction (XRD), which confirms the crystalline structure; scanning electron microscopy (SEM) to reveal surface morphology, transmission electron microscopy (TEM), to examine the nanoscale dispersion, and thermal analysis, to asses the material stability. Various experimental parameters, including pH and catalyst mass loading, were tuned. The adsorption data were found to be best described by Langmuir isotherm, indicating monolayer adsorption. The reaction order was found to obey pseudo-first-order kinetics, suggesting that physisorption controls the process. The synthesized catalyst had a retention efficiency of 92% across three consecutive cycles, demonstrating its potential as a reusable and efficient adsorbent for Cu(II) removal from water.

**Supplementary Information:**

The online version contains supplementary material available at 10.1038/s41598-025-96777-y.

## Introduction

Heavy metal contamination in water, including copper (Cu), lead (Pb), mercury (Hg), and cadmium (Cd), poses a significant risk to both human health and the environment. These metals can accumulate in water sources and are extremely harmful, even in low concentrations. They are often found in industrial waste, mining runoff, and agricultural practices^1–4^. Copper is a necessary trace metal for all living organisms; however, excessive levels can harm human health and aquatic ecosystems. Heavy metal contamination in water sources represents a major environmental issue, largely attributable to industrial activities^5,6^. Mining and smelting industries significantly contribute to the release of heavy metals, including copper, into adjacent water bodies^7,8^. Mining operations have contributed to water contamination with heavy metals in several regions, notably the Tar Creek area in northeast Oklahoma, which has experienced significant pollution from extensive lead and zinc mining activities^9,10^.

The exposure of contaminated water, copper can lead to neurological and liver dysfunction, harm organs, and interfere with essential biological processes^11–15^. Once these metals enter the water, they tend to settle in sediments, making it difficult to remove using traditional methods, which causes bioaccumulation in the food chain. To effectively mitigate heavy metal pollution and protect water quality, it is essential to develop advanced filtration systems and adsorbent materials, such as chitosan^16–19^.

Conventional techniques for removing heavy metals include adsorption, extraction, oxidation-reduction, and electrochemical methods^20^. However, due to the use of imported extractants during extraction, the process of removing heavy metals from soil or residues may contaminate groundwater or nearby water sources^21,22^.

The oxidation-reduction process typically produces waste residues^23^. Because of the poor removal, high power cost, and metal selectivity of the electrochemical approach, its application to removing heavy metals from water is restricted^24^. Adsorption is widely utilized because of its many benefits, including the low cost of the adsorbent, ease of control, and high removal effectiveness^25,26^. Nano-carbon, nanoscale metal oxide, maghemite, dendrimers, and alginate nanoparticles are the conventional nano-adsorbents used to remove Cu(II) and As (III)^27,28^. Unfortunately, the time it takes to separate adsorbents from absorbents and the high cost of separation accompany most nano-adsorbents, severely restricting their applicability^29,30^. Consequently, adsorption of heavy metals by nano-size based materials is extensively mentioned in literature^31,32^.

Chitosan, a biopolymer derived from chitin, has exhibited potential for environmental applications due to its biodegradability, abundance, and inherent properties that facilitate metal ion binding^33–39^. The exoskeletons of crustaceans and insects contain chitin, the second most prevalent biopolymer after cellulose^40–42^. Chitosan is produced by deacetylating chitin, which produces a polymer with functional amino and hydroxyl groups^43–45^. These functional groups are highly effective for various adsorption processes because they can form complexes with metal ions through ion exchange, chelation, and electrostatic attraction^42,46,47^. The literature referenced the extensive adsorption of copper ions onto Chitosan surfaces. Verbych et al.^48^reported the adsorption capacity of Chitosan to be 1.8–2.2 mmol/g of dried mass in the pH range of (5–6).

In the chemical composition of XY_2_O_4_, metal oxides are characterized by having a spinel structure. In this structure, Y represents a trivalent metal ion at the octahedral position, while X represents a divalent metal ion at the tetrahedral position. Additionally, oxygen anions are grouped in a close-packed cubic array^49–53^. Spinel Oxides have attracted substantial attention as effective adsorbents for removing copper (Cu²⁺) ions from contaminated water^54,55^. The presence of spinel oxide structure enhances the chemical stability along with the adsorption capacities by providing abundant active sites, increasing surface area, and facilitating electron transfer.

The preparation of spinel oxides often requires energy-intensive methods such as high-temperature calcination, hydrothermal synthesis, or sol-gel processes. These techniques can be costly and difficult to scale up while maintaining uniform particle size and adsorption efficiency.

Another major limitation is regeneration and reusability. While spinel oxides exhibit high adsorption capacities, their ability to be efficiently regenerated and reused in multiple cycles is often compromised. Regeneration typically involves chemical desorption using strong acids or bases, which can lead to partial degradation of the material, structural changes, or loss of adsorption capacity over time.

Spinel Oxide and Chitosan were found to facilitate the adsorption of copper ions. The amine (-NH₂) and hydroxyl (-OH) groups of Chitosan can significantly increase the Cu(II) adsorption/exploitation rate compared to NiCo₂O₄, mainly due to their strong affinity for metal ions through chelation, electrostatic attraction, and hydrogen bonding. Wang et al.^56^ reported the development of a magnetic microsphere-MnFe_2_O_4_/Chitosan composite to remove copper ions. The maximal adsorption capacity of Cu(II) was 22.6 mg /g.

In this work, Spinel Oxide was prepared and loaded on the surface of Chitosan to enhance copper ions’ adsorption in a water sample. Comparative studies were performed for Pristine Spinel Nickel Cobaltite and Chitosan-modified composites. The adsorption mechanism was evaluated using various models, such as Langmuir and Freundlich. The order of reaction was estimated for modified and pristine Chitosan.

## Experimental section

### Chemical and reagents

Chitosan, Nickel sulfate, Cobalt acetate, Ammonium hydroxide, Ethanol, Copper sulfate, Sodium tartrate, Hydrochloric acid, and Sodium hydroxide were used without further purification, exactly as received. All of the chemicals were sourced from Sigma-Aldrich and are analytical grade. The solutions were made using double-distilled water.

### Preparation of nickel Cobalt spinel oxide

The following procedure was used to produce the NiCo_2_O_4_: Initially, 25 mL of Ethanol was mixed with 0.873 g of Nickel nitrate and 1.746 g of Cobalt nitrate, and the mixture was agitated for 5 min. Then, propylene oxide was added drop-wise as gelation agent till the mixture was agitated for four hours. Then, it was stirred for two more hours at 70 ^o^C. Finally, it was calcined for four hours at 600 °C.

### Preparation of NiCo_2_O_4_ supported on Chitosan

NiCo_2_O_4_@Chitosan composite was synthesized using the following procedure^57^: First, 1 g of NiCo_2_O_4_ powder was suspended in 100 mL of 1% Acetic Acid (pH $\:\sim$ 3). Next, 1 g of Chitosan was added to the solution, which was then sonicated for 30 min. The pH of the solution was subsequently adjusted to be neutral (pH ~ 6) using 0.1 M NaOH. Finally, the catalyst was filtered, washed, and dried at 50 °C for 6 h. The drying conditions on the Chitosan adsorption efficiency and moderate temperature used in the drying process are necessary to prevent the collapse of a porous structure crucial for the adsorption process.

### UV-Vis measurement

UV–Vis Thermo Fischer Scientific Model (Evolution 60) ranged from 200 to 900 nm. The detection of copper sulfate concentration was measured at a wavelength of 230 nm. Detection of UV-Vis spectra was investigated using Vision lite software Ver 2.2.

To evaluate the adsorption properties of Chitosan and NiCo_2_O_4_@Chitosan for Copper (II), adsorption tests were conducted at varying pH values (3, 4, 5, and 6) and initial concentrations 400 mg/L. For the kinetics study, 5 mg/mL samples of Chitosan and NiCo_2_O_4_@Chitosan were added to an aqueous solution containing Copper (II), with an initial concentration set at 400 mg/L. The copper solution was obtained from a 0.1 M copper sulfate stock solution. To maintain the desired pH levels during the testing, 0.1 mol/L solutions of NaOH or HCl were used. The final results reported are the mean values obtained from three tests to minimize errors. The quantity of Copper (II) adsorbed at a given contact time (q_me_) was calculated using the following equation:

1 $${\text{Q}}_{{{\text{me}}}} = \:\frac{{C_{{bc}} - C_{e} }}{M}{\text{V}}$$

In this equation, *C*_bc_ and *C*_e_ (in mg/L) represent the concentrations of copper (II) in the initial and equilibrium liquid phases, respectively; V(L) is the volume of the solution, and M(g)is the mass of dry Chitosan and NiCo_2_O_4_@Chitosan.

## Results and discussion

### Analysis

**Fig. 1:**
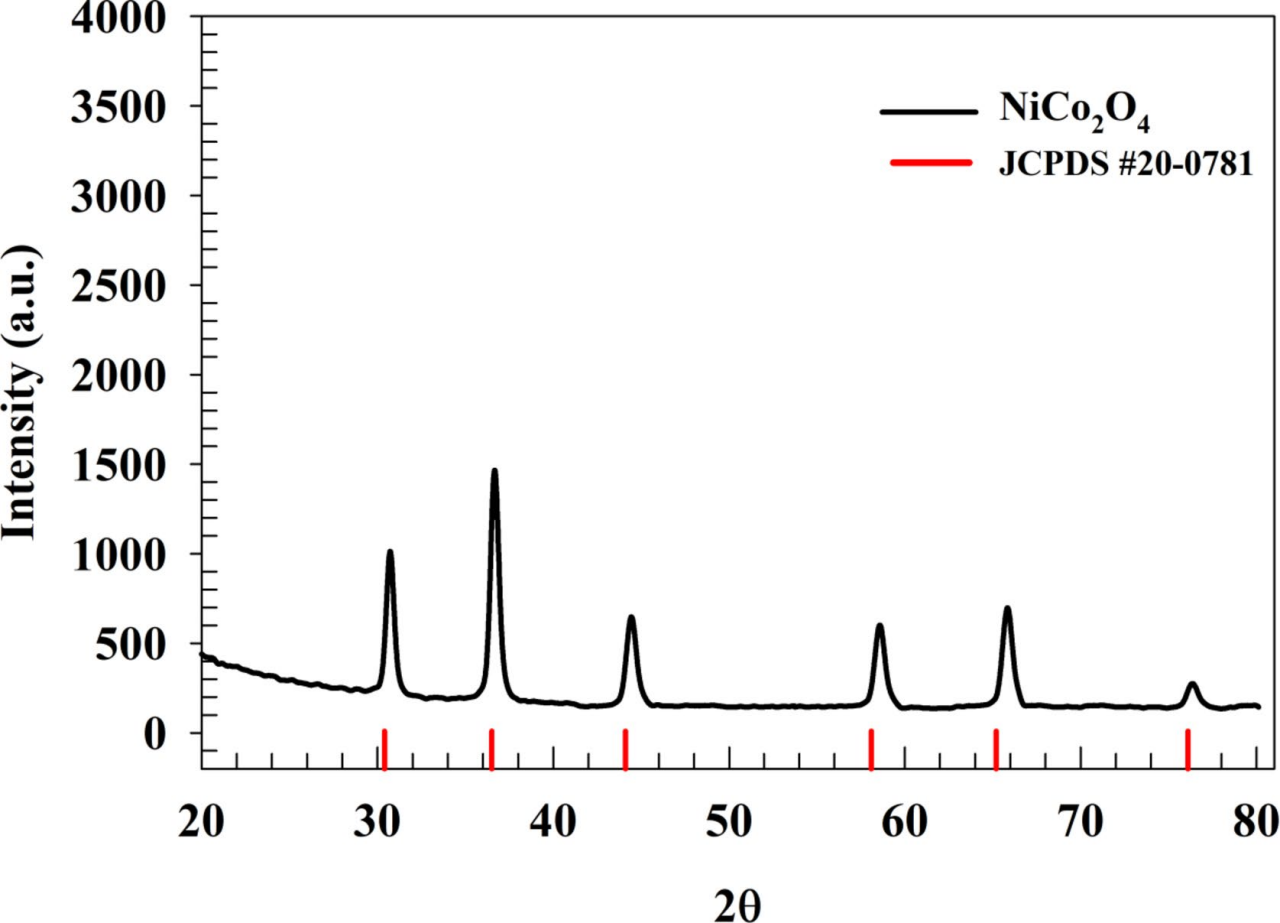
XRD pattern of NiCo_2_O_4_.

Powder X-ray diffraction (XRD) was used to evaluate the chemical structure of the Nickel cobaltite combined with Chitosan. By comparing the diffraction peaks of crystalline NiCo_2_O_4_@Chitosan with the amorphous peaks of m-C (JCPDS no. 20–0781)^58^, the XRD pattern is illustrated in Fig. 1. This analysis confirms the presence of the spinel NiCo_2_O_4_ structure. The hkl planes of {220}, {311}, {400}, {511}, {440}, and {533} correspond to peaks at 2θ values of 30.3°, 36.4°, 44.3°, 58.3°, 65.1°, and 76.3°, respectively^59,60^. The XRD result is attributed to a cubic crystal system with a point group of m3m. Additionally, a hump observed at approximately 2θ ~ 12.6° is attributed to the Chitosan sheets^61,62^.

**Fig. 2:**
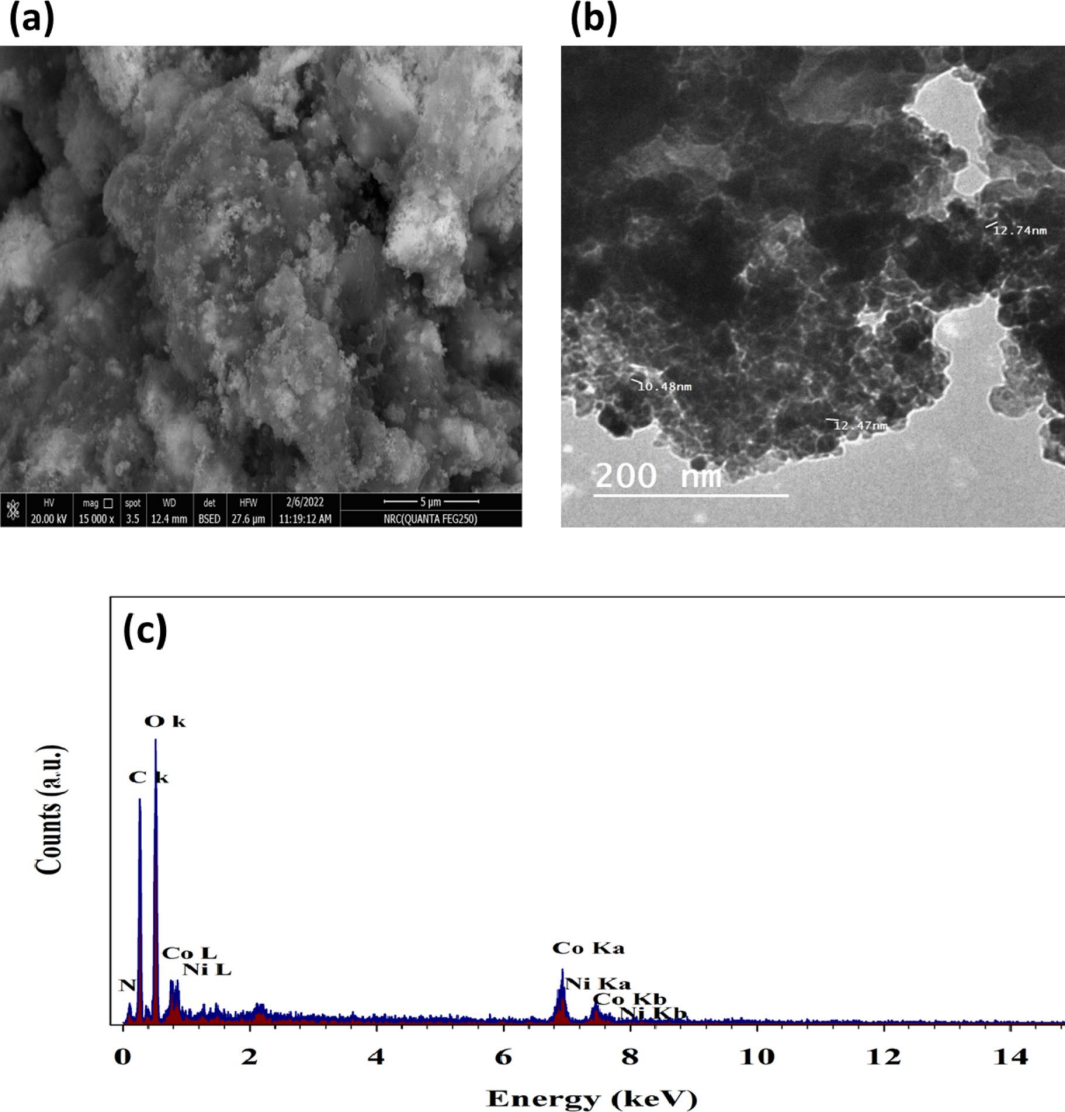
(**a**) SEM, (**b**) TEM of NiCo_2_O_4_@Chitosan sample. (**c**) EDX of NiCo_2_O_4_@Chitosan surface.

An examination of the morphological properties of NiCo_2_O_4_ nanoparticles was conducted using scanning electron microscopy (SEM), as illustrated in Fig. 2a. The particle size distribution was found to range from 35 to 80 nanometers. The small size of the Nickel Cobaltite particles suggested a higher level of chemical activity. Elsemore, smaller NiCo₂O₄ particles can improve binding with the matrix, leading to increased hardness and durability. Figure 2a also shows how Nickel Cobaltite is incorporated into the Chitosan sheets, demonstrating that the particles are evenly dispersed.

Moreover, Transmission Electron Microscopy was employed to ascertain the shape of NiCo_2_O_4_nanoparticles.

TEM images of NiCo_2_O_4_@Chitosan composites are displayed in Fig. 2b. The mean particle diameter of these composites is estimated to be between 20 and 50 nm. Based on the findings of the investigation, it was discovered that the nanospheres made of NiCo_2_O_4_ showed signs of attachment to the sheets of Chitosan. The presence of NiCo_2_O_4_ on the Chitosan sheet is validated by utilizing the appropriate transmission electron microscopy (TEM) diffraction patterns. The elements Ni, Co, O, C, and N were verified to be the primary constituents of NiCo_2_O_4_@Chitosan by the EDX spectra (Fig. 2c). The EDX was employed for determination of elemental ratios. Thus, the provided amount of cobalt to nickel was equal to two.

**Fig. 3:**
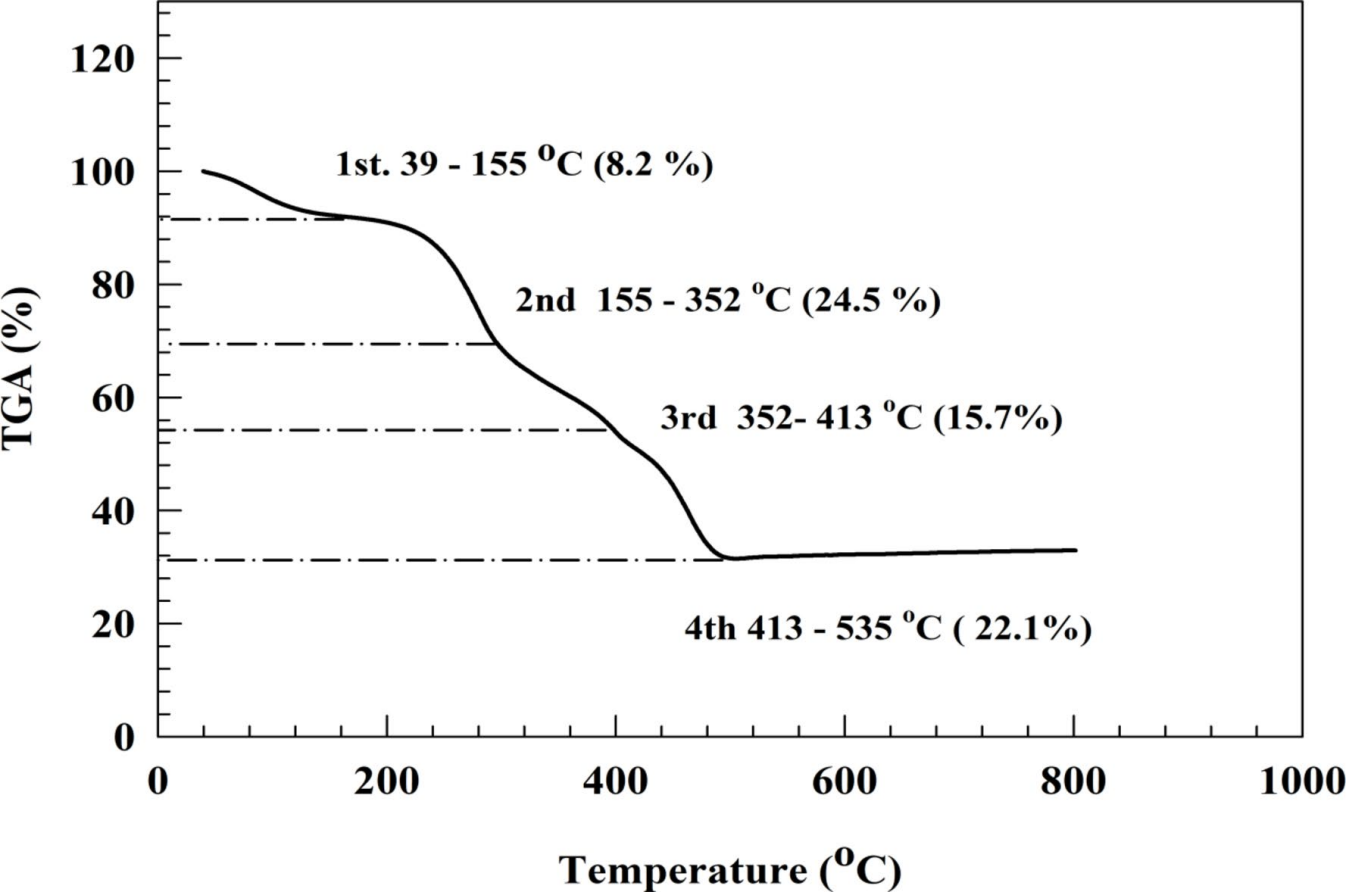
TGA of NiCo_2_O_4_@Chitosan.

Thermal analytical technique (TGA) was utilized in order to determine the thermal durability and chemical degradation of the material. Figure 3illustrates the TGA of the NiCo_2_O_4_@Chitosan. The composite exhibited four thermal transitions between temperatures of 80.1, 279.3, 394.1, and 460.1 degrees Celsius, with weight loss percentages of 8.2%, 24.5%, 15.7%, and 22.1%, respectively. The elimination of water is responsible for the thermal change that occurs at approximately 80 ^o^C. Furthermore, it has been noted that the heat degradation of Chitosan is a process that occurs in two stages^63^. Consequently, the thermal decomposition peaks at 279.3 and 394.1^o^C are anticipated to occur during the breakdown of Chitosan. The fourth thermal transition occurs in the temperature range of 460.1 ^o^C due to the conversion of residuals of NiCo_2_O_4_ that converted to corresponding hydroxide during preparation of NiCo_2_O_4_@Chitosan in alkaline medium. Additionally, the chemical structure of Chitosan was confirmed using FT-IR spectra, as represented in Fig. S1.

### Study of copper adsorption on Chitosan and NiCo_2_O_4_@Chitosan

**Fig. 4:**
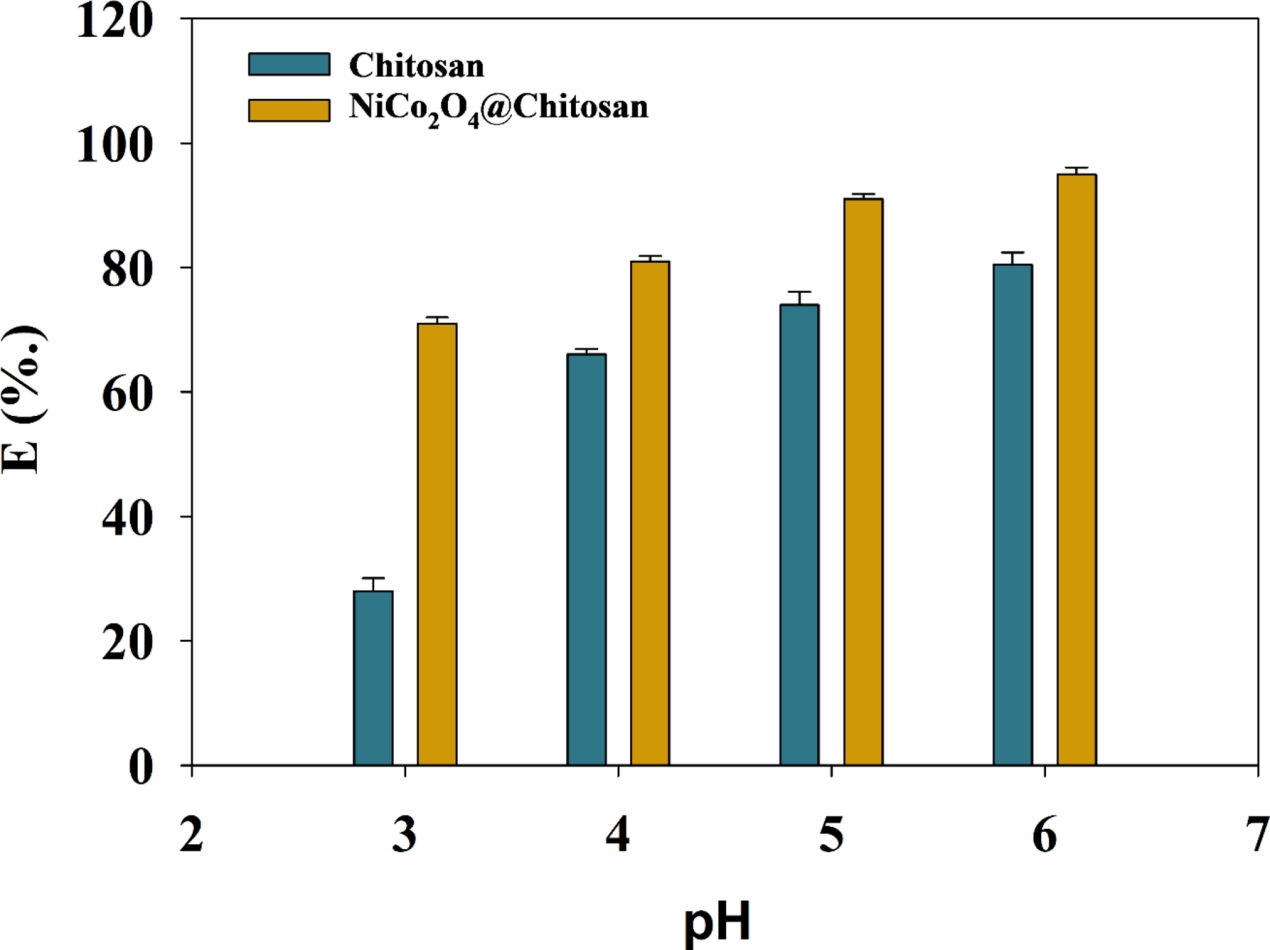
Relation between pH and efficiency of Cu(II) removal.

**Fig. 5:**
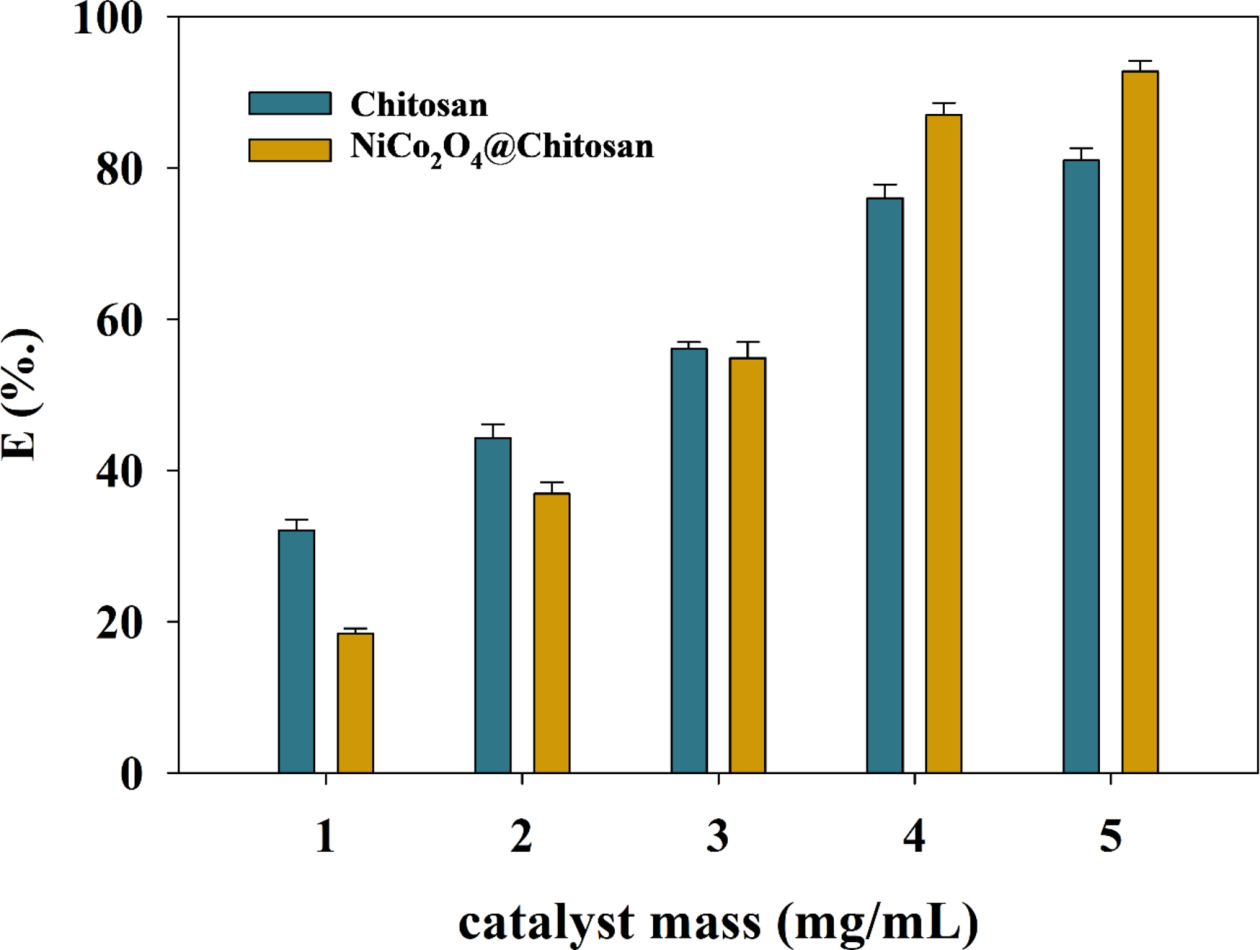
Relation between catalyst loading and efficiency of Cu(II) removal.

The adsorption capacity and the process as a whole are greatly impacted by the pH of the solution. Using a 4-hour incubation time at 25 °C, the study examined the adsorption properties of Cu(II) ions at different initial pH values. The fact that Chitosan’s nitrogen atom has an extra pair of electrons suggests that the NH_2_ groups are necessary for heavy metal adsorption onto the surface of material. Using a fixed starting concentration of Cu(II) ions at 400 mg/L over a pH range of 3 to 6, the study investigated the impact of pH on the adsorption properties of Cu(II) ions onto Chitosan and NiCo_2_O_4_@Chitosan composites. The beneficial impact of the initial pH on the two types of adsorbents’ adsorption effectiveness is seen in Fig. 4. Due to a high concentration of H^+^ ions, the hydroxyl and amine groups of Chitosan undergo protonation at a pH of 3. There are fewer binding sites available for Cu(II) ions because of protonation, which occurs when protons (H^+^) in the aqueous solution compete with heavy metal ions for active adsorption sites on Chitosan and NiCo_2_O_4_@Chitosan composites. The generated composite’s surface has a negative charge at pH values higher than 5.3, which increases the ability of heavy metal ions to adsorb onto the Chitosan-based surfaces. Copper precipitates out of the solution as copper hydroxide when the pH level rises. In a Copper sulfate solution with a pH of 6, the impact of catalyst loading was examined at doses ranging from 1 to 5 mg/mL. By evaluating the adsorption effectiveness, the catalyst mass was determined. The relationship between Chitosan mass and NiCo_2_O_4_@Chitosan is shown in Fig. 5.

**Fig. 6:**
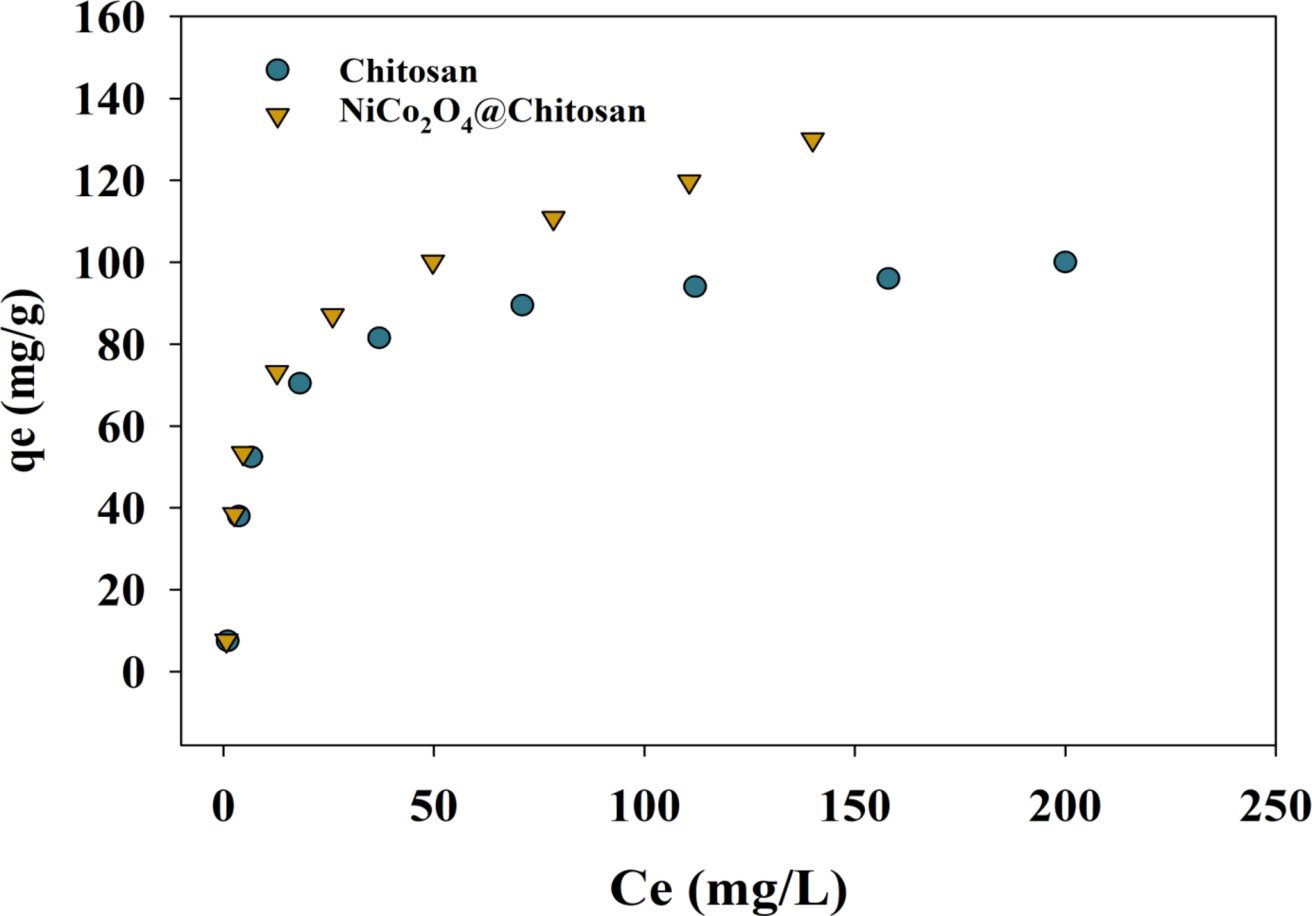
Adsorption isotherms of Cu(II) on Chitosan and NiCo_2_O_4_@Chitosan composite.

Adsorption systems are characterized by the presence of the equilibrium adsorption isotherm, which plays a vital part in the process of comprehending the interaction that occurs between the adsorbent and the adsorbate. As shown in Fig. 6, the relationship between the adsorption equilibrium concentration (Ce) and adsorption capacity (qe, ). Whereas, the qe of both metal ions increased with concentration till the equilibrium was achieved at around 200 mg/L.

The equilibrium adsorption isotherms of Cu(II) on the synthesized Chitosan and NiCo_2_O_4_@Chitosan adsorbents were analyzed using the Freundlich and Langmuir adsorption models to provide a thorough grasp of the isotherms and their characteristics. The Freundlich adsorption model assumes that adsorption takes place on heterogeneous surfaces, enabling the examination of multilayer adsorption phenomena. In contrast, the Langmuir adsorption model suggests that adsorption takes place at distinct and homogenous sites inside the adsorbent material^31^. The Eqs. (2) and (3) provide a linearization of the Langmuir and Freundlich models.

2 $$\:\frac{{C_{e} }}{{q_{e} }} = \:\frac{{C_{e} }}{{q_{m} }} + \:\frac{1}{{k_{L} q_{m} }}$$

3 $${\text{Log}}\:q_{e} = {\text{Log}}\:K_{F} + \:\frac{1}{n}{\text{Log}}\:C_{e}$$

Where K_L_ is Langmuir adsorption constant, qe and qm are adsorption capacity and the maximum adsorption capacity, respectively, Ce is the equilibrium heavy metal concentration, K_F_ is adsorption capacity, n is adsorption intensity. Therefore, the constants can be estimated by plotting relation between Ce/qe vs. Ce. Thus, plot’s slope and y-intercept serve as indicators of K_L_ and qm, respectively. Additionally, by studying the linear relation between the logqe and Ce, the values of n and K_F_ can be recognized. By evaluating the connection between the two variables, this can be accomplished.

**Fig. 7:**
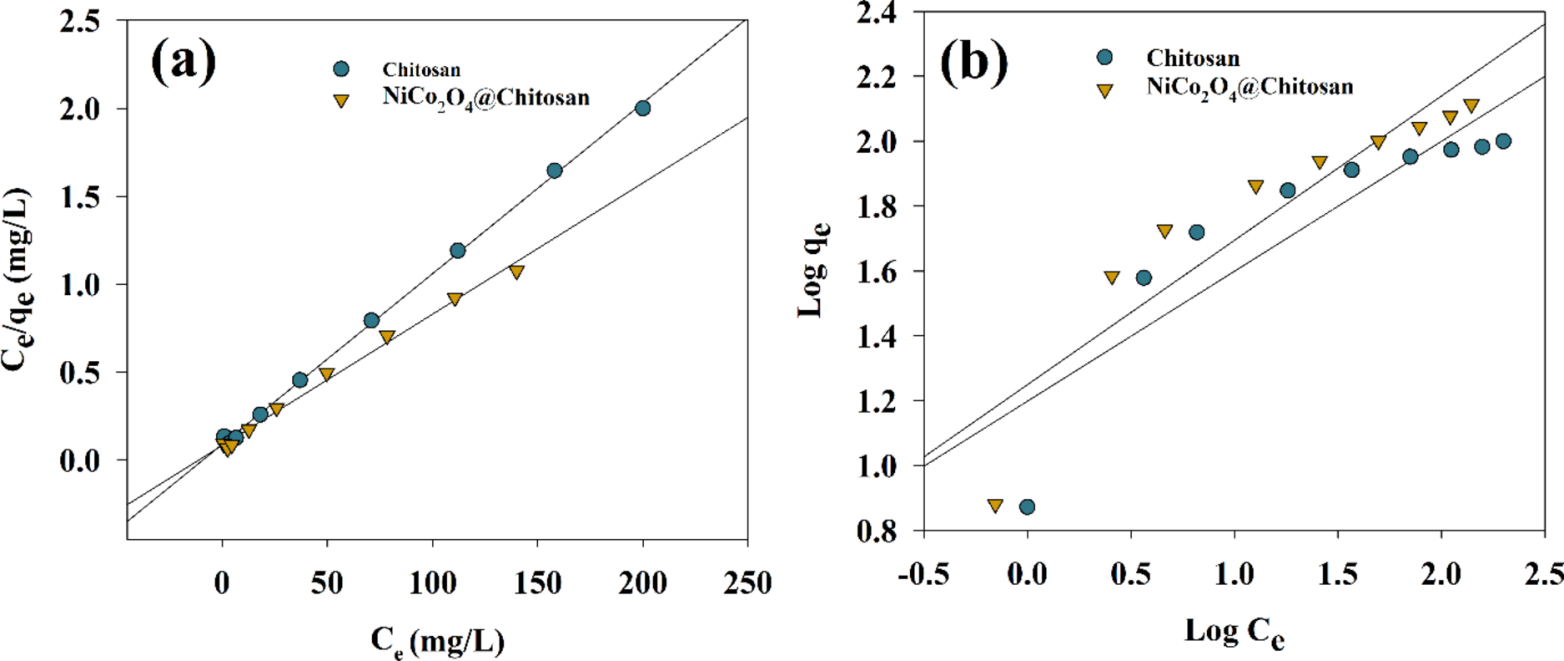
Adsorption for Cu(II) ions using (**a**) Langmuir and (**b**) Freundlich models (pH = 5, t_contact_ = 4 h).

The graphs of the two models are shown in Fig. 7, and the parameters of Cu(II) adsorption are listed in Table 1. Given its higher correlation coefficient, the monolayer Langmuir adsorption isotherm is thought to be better suited to explaining the adsorption seen on the produced Chitosan and NiCo_2_O_4_@Chitosan composite. Furthermore, the Langmuir model’s estimated qe value closely matches the outcomes of the experimental trials. The Chitosan and NiCo_2_O_4_@Chitosan composite demonstrated a maximum adsorption capacity of 134.9 mg/g for Cu(II) in accordance with this model. Table 1 summarizes the findings of several investigations that were carried out to compare the adsorption capacities of Cu(II) adsorbates. Significantly improved performance is shown when comparing the adsorption capacities of Chitosan and the NiCo_2_O_4_@Chitosan composite with those of other adsorbents. The Langmuir isotherm and Freundlich isotherm describe different adsorption behaviors. The better fit of the Langmuir model suggests that the adsorption process in your study follows monolayer adsorption on a homogeneous surface rather than multilayer adsorption on a heterogeneous surface.

**Table 1:** Langmuir and Freundlich parameters for Cu(II) adsorption onto Chitosan and NiCo_2_O_4_@Chitosan adsorbents.

Adsorbent	Langmuir model			Freundlich model		
	q_max_ (mg/g)	K_L_ (L/mg)	*R* ^2^	*N*	K_F_ (mg)^1^^-^^1/^^n^(L)^1^^/^^n^ (g)^−1^	*R* ^2^
Chitosan	103.1	0.10888	0.9988	2.5	2.71	0.7874985
NiCo_2_O_4_@Chitosan	134.9	0.08979	0.9925	2.3	2.71	0.8473552

**Fig. 8:**
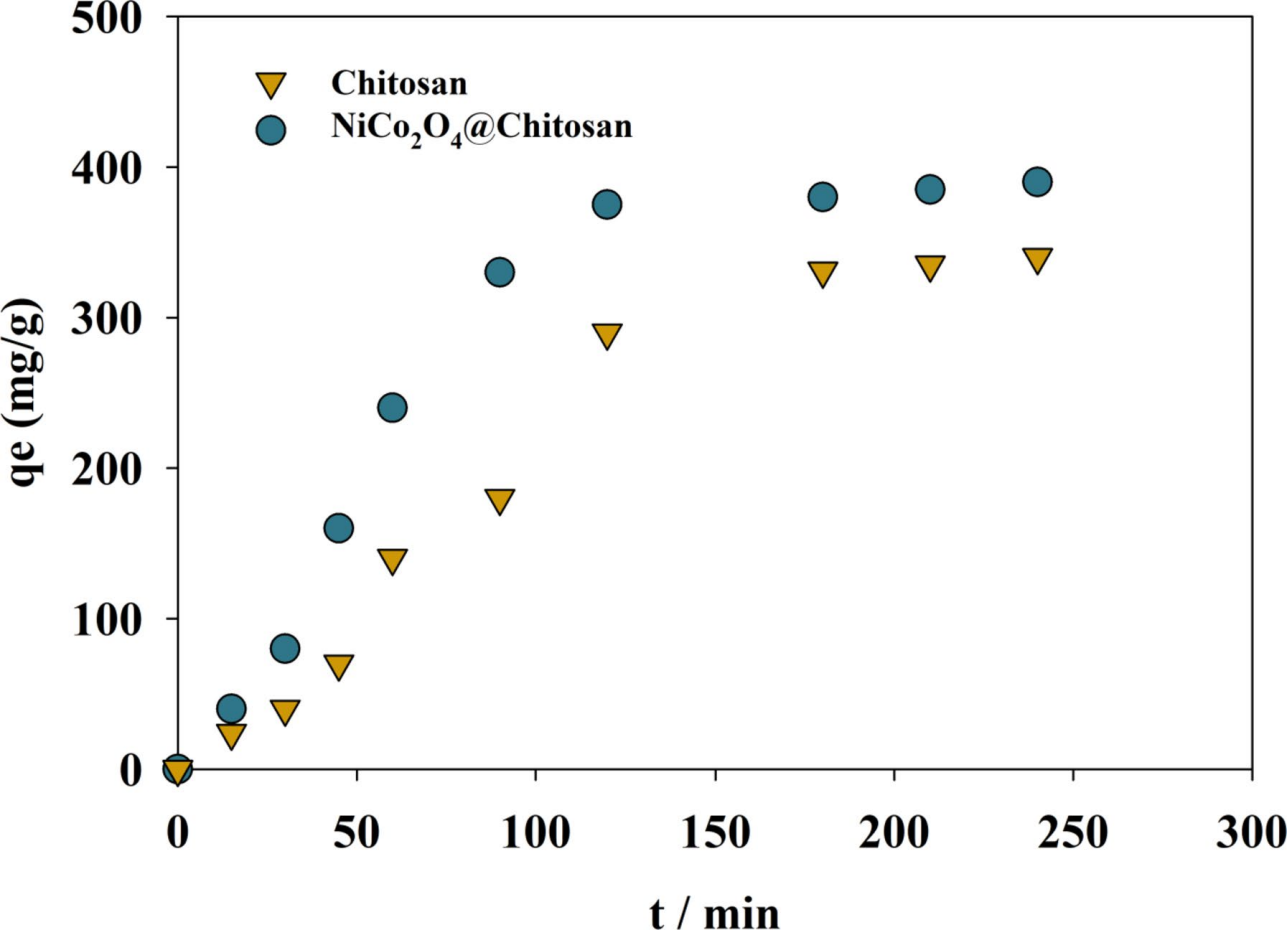
The relation between qe and time for different Chitosan and NiCo_2_O_4_@Chitosan composites.

**Fig. 9:**
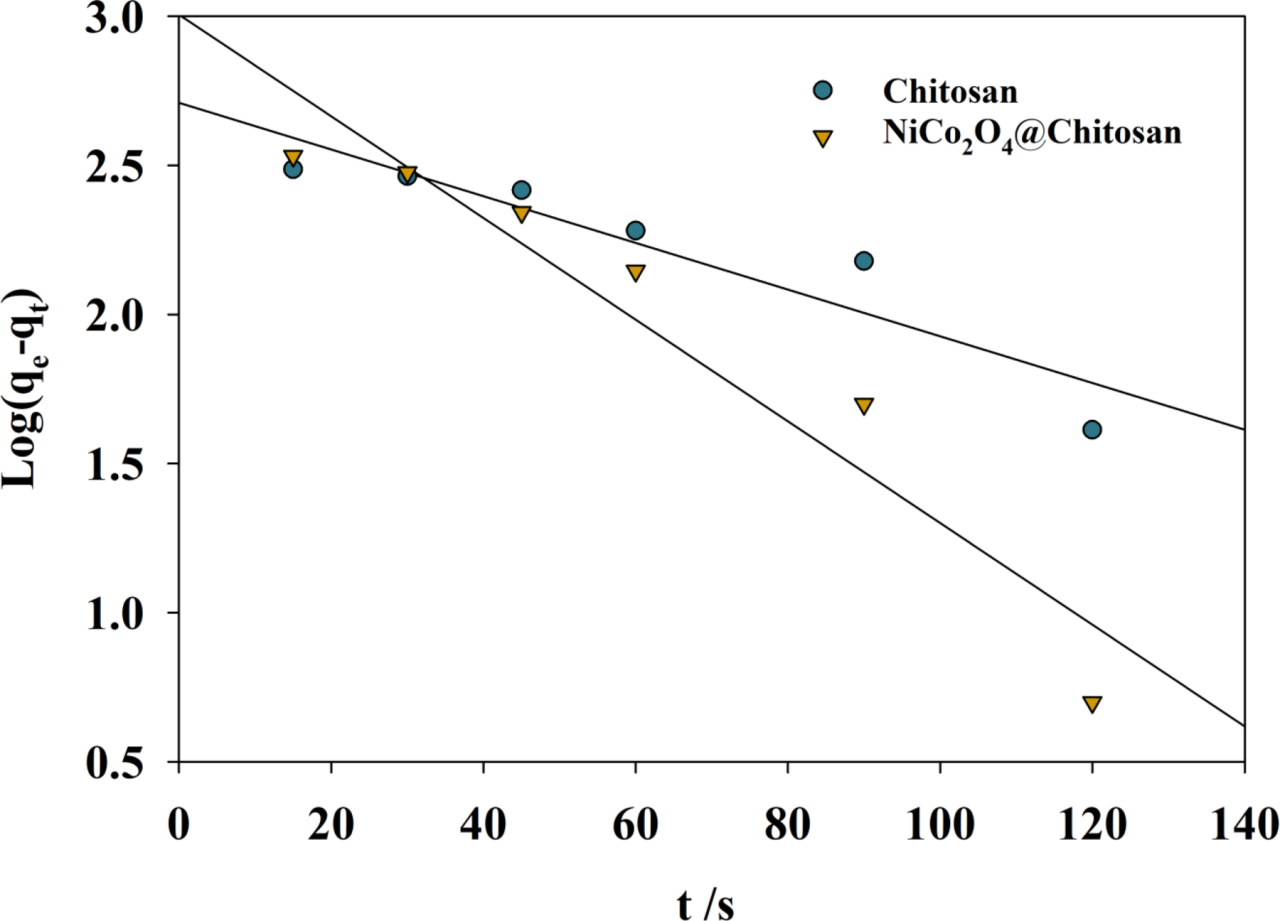
Pseudo-first-order model for Cu-adsorption upon Chitosan and NiCo_2_O_4_@Chitosan surfaces.

The adsorption of Cu(II) and Cd(II) was examined during 360 min to better understand the adsorption process’s kinetics. The impact of contact time on Cu(II) adsorption onto the composite of Chitosan and NiCo_2_O_4_@Chitosan is depicted in Fig. 8. During the first 40 min of contact, the adsorption of Cu(II) ions increased rapidly in both the Chitosan and NiCo_2_O_4_@Chitosan composite, reaching equilibrium after 120 min. A concentration differential between the adsorbate in the solution and the adsorbent surface causes a high adsorption rate during the first phase of adsorption. However, because accessible sites on the adsorbent are occupied, the adsorption rate drops from 120 to 250 min as the concentration of Cu(II) ions steadily drops. Different adsorption techniques’ effectiveness in eliminating organic and inorganic materials from water and non-aqueous environments may be assessed using kinetics. Accurate adsorption predictions during dissolved chemical extraction are also made possible by kinetic analysis. Furthermore, comprehending the adsorption process requires a grasp of kinetics. The commonly used pseudo-first-order model was used to evaluate the NiCo_2_O_4_@Chitosan composite’s ability to efficiently adsorb copper from water (see Fig. 9). The linear form of the pseudo-first-order equation can be represented as follows:

4 $$\:{\text{Log(qe}} - {\text{qt) = Log(qe) }} - \frac{{K_{1} t}}{{2.303}}$$

The values (qe) and (qt) (mg/g), which stand for the equilibrium state and the quantity adsorbed at a certain time (t) (min), respectively, indicate the adsorption capacities of the Chitosan and NiCo_2_O_4_@Chitosan composite. Furthermore, the symbol (k1), expressed in min^− 1^, denotes the pseudo-first-order model’s rate constant. Nonetheless, there exists no association between the actual (qe) values and the calculations derived from the pseudo-first-order equation (see Table 2). This difference indicates that mass transport in the solution minimally influences the adsorption process, which is predominantly governed by chemisorption^64^.

**Table 2:** The parameters of the pseudo first-order model for the adsorption of Cu(II).

Surface	Pseudo first order kinetic model		
	k1 (mgg^− 1^)	qe (mgg^− 1^)	*R* ^2^
Chitosan	0.018424	504	0.9180921
NiCo_2_O_4_@Chitosan	0.039151	630	0.9642065

### Adsorbent recycling

**Fig. 10:**
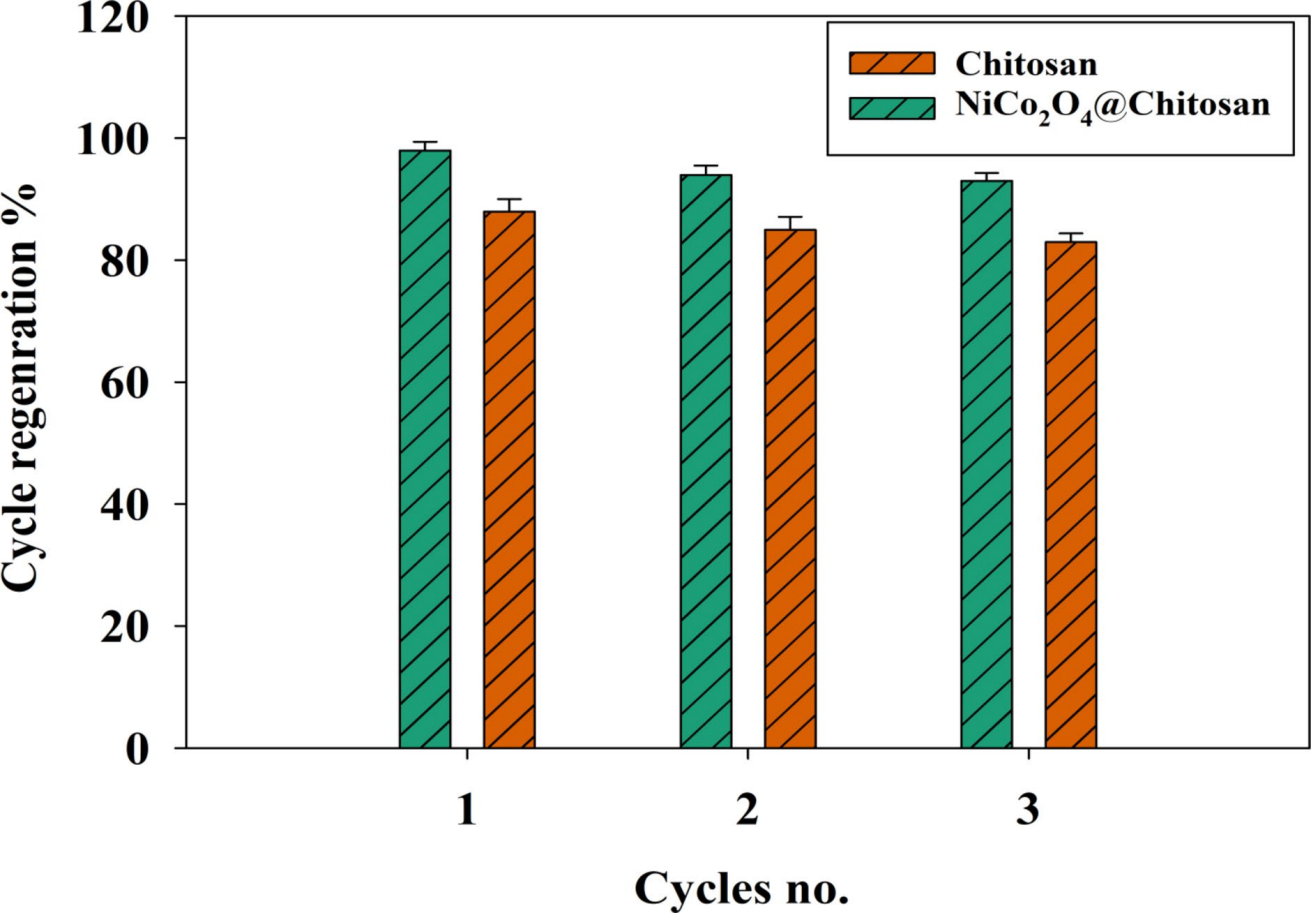
The efficiency of Chitosan and NiCo_2_O_4_@Chitosan adsorbents for the adsorption of Cu for three cycles.

The regeneration process influences the adsorption capacity. Adsorption capacity decreases gradually with repeated use, depending on how well the adsorbent withstands the regeneration conditions. An analysis of the adsorption/elution and regeneration process indicates that the necessary acid concentration is crucial for economic viability and the reusability of the adsorbent. The suitability of 0.10 M HCl for achieving maximum elution of adsorbed metal ions from the adsorbent has been confirmed. Following the elution process, the adsorbent is restored to its original state for subsequent adsorption experiments. In every cycle experiment, the concentration of metal ions was set at 400 mg L^− 1^, with 0.005 g of adsorbent utilized in a 50 mL beaker and a 10 mL HCl solution employed as the eluent. Figure 10 illustrates that following three adsorption cycles, the adsorption capacities of Cu diminished to 83% and 93% for Chitosan and NiCo_2_O_4_@Chitosan, respectively. The data indicate that the adsorption efficiency of the adsorbent after 3 cycles exceeds 80% reversibility, demonstrating that Chitosan and NiCo_2_O_4_@Chitosan, adsorbent is an environmentally friendly and sustainable option for metal ion adsorption. Otherwise, using 0.1 M HCl for regenerating adsorbents has some limitations for Chitosan and Chitosan/NiCo_2_O_4_ composite. For instance, incomplete Desorption of Cu(II) ions due to limited effectiveness on NiCo₂O₄, and Potential Degradation of Chitosan by partially soluble in acidic environments.

## Conclusion

Integrating Nickel Cobaltite with biopolymers such as Chitosan can provide hybrid adsorbents with improved efficacy. These innovative materials provide an environmentally sustainable and cost-effective solution for remediating water contaminated with copper and other heavy metals, presenting a feasible approach to addressing water pollution in industrial and environmental settings. Research aimed at improving the manufacturing and effectiveness of Nickel Cobaltite-based adsorbents continues to yield significant progress in water treatment technologies. The adsorption of copper using NiCo_2_O_4_@Chitosan was investigated within the range of pH (3–6) due to the limitation of Chitosan use in low pH. Otherwise, the precipitation of copper is at a high pH. The provided rate constants are 0.018424 and 0.039151 (mg/g) for Chitosan and NiCo_2_O_4_@Chitosan, respectively. Additionally, NiCo_2_O_4_@Chitosan will be employed to remove other heavy metals from a water sample. Whereas, the Chitosan functionalized with nanoparticles showed promising results in heavy metal adsorption. The fit of the Langmuir model indicates that the adsorption process in your study follows monolayer adsorption on a uniform surface rather than multilayer adsorption on a varied surface.

## Electronic supplementary material

Below is the link to the electronic supplementary material.

Supplementary Material

## Data Availability

The datasets used and/or analysed during the current study are available from the corresponding author on reasonable request.
