# Supplementary material for: Synthesis of NiCo2O4 supported on Chitosan for potential adsorption of copper ions in water samples
Source: Sci Rep. 2025 Apr 24;15:14402. doi: 10.1038/s41598-025-96777-y (PMC12022117; doi:10.1038/s41598-025-96777-y)
Supplement: Supplementary file 1 — Supplementary Material [file 41598_2025_96777_MOESM1_ESM.docx]

**1.Physical Measurements**

Powder X-ray diffraction (PXRD) patterns were recorded at room temperature on a Bruker D8 Advance AXS diffractometer with Cu Kα radiation (λ = 1.54178 Å) and a Goebel mirror in θ−2θ geometry with a position-sensitive detector in a 2θ range from 5° to 50° at a scan speed of 1° min^−1^. α-Al_2_O_3_ was employed as external standard. The powder samples were filled into glass capillaries (diameter = 0.7 mm). Each capillary was sealed prior to the measurement.

UV–Vis spectrophotometric measurements were carried out using automated spectrophotometer UV–Vis Thermo Fischer Scientific Model (Evolution 60) ranged from 200 to 900 nm. The detection of copper sulfate concentration was measured at a wavelength of 230 nm. Detection of UV-Vis spectra was investigated using Vision lite software Ver 2.2. Thermal analyses were carried out using a Shimadzu TGA-50H and DTA-50H thermogravimetric analyzer in a dynamic nitrogen atmosphere (flow rate 20 ml min^-1^) with a heating rate of 10°C min^-1^. The percentage weight loss was measured from ambient temperature to 600 °C, highly sintered a-Al_2_O_3_ was used as reference. The Quanta FEG 250 (USA) equipment and an EDAX Unit (Energy Dispersive X-ray Analyses) connected to a SEM with an accelerating voltage of 30 kV, magnification of 14 up to 1000000, and resolution for Gun were used to examine the samples' microstructure. Furthermore, the structure of NiCo_2_O_4_@Chitosan was estimated using transmitted electron microscope by [JEOL JEM-1400](https://research.missouri.edu/jeol-jem-1400) instrument.

**2. FT-IR analysis for chitosan:**

Several different absorption bands were discovered in the FTIR spectrum of chitosan, which allowed for the identification of the unique functional groups that were observed in the mid-infrared range of 4000–400 cm−1. Chitosan's infrared spectrum is depicted in **Figure S1**. It was determined that the stretching vibrations of the O–H bond in the produced chitosan were measured at a frequency of 3445.44 cm^−1^. At a frequency of 2937.41 cm^−1^, the C–H transition was identified. C=O stretching of the amide I band is responsible for the absorption peaks that occur at 1635.84, 1571.05, 1434.48, and 1370.84 cm^−1^. These peaks are characterized by the bending of the N–H, C–H, and O–H, respectively[1–3]. Anti-symmetric stretching of the (C–O–C) bridge was ascribed to the peak at 1159.45 cm^−1^, whereas skeletal vibrations involving C–O stretching were projected to occur at 1085.43 cm^−1^ and 1022.32 cm^−1^, respectively[4].


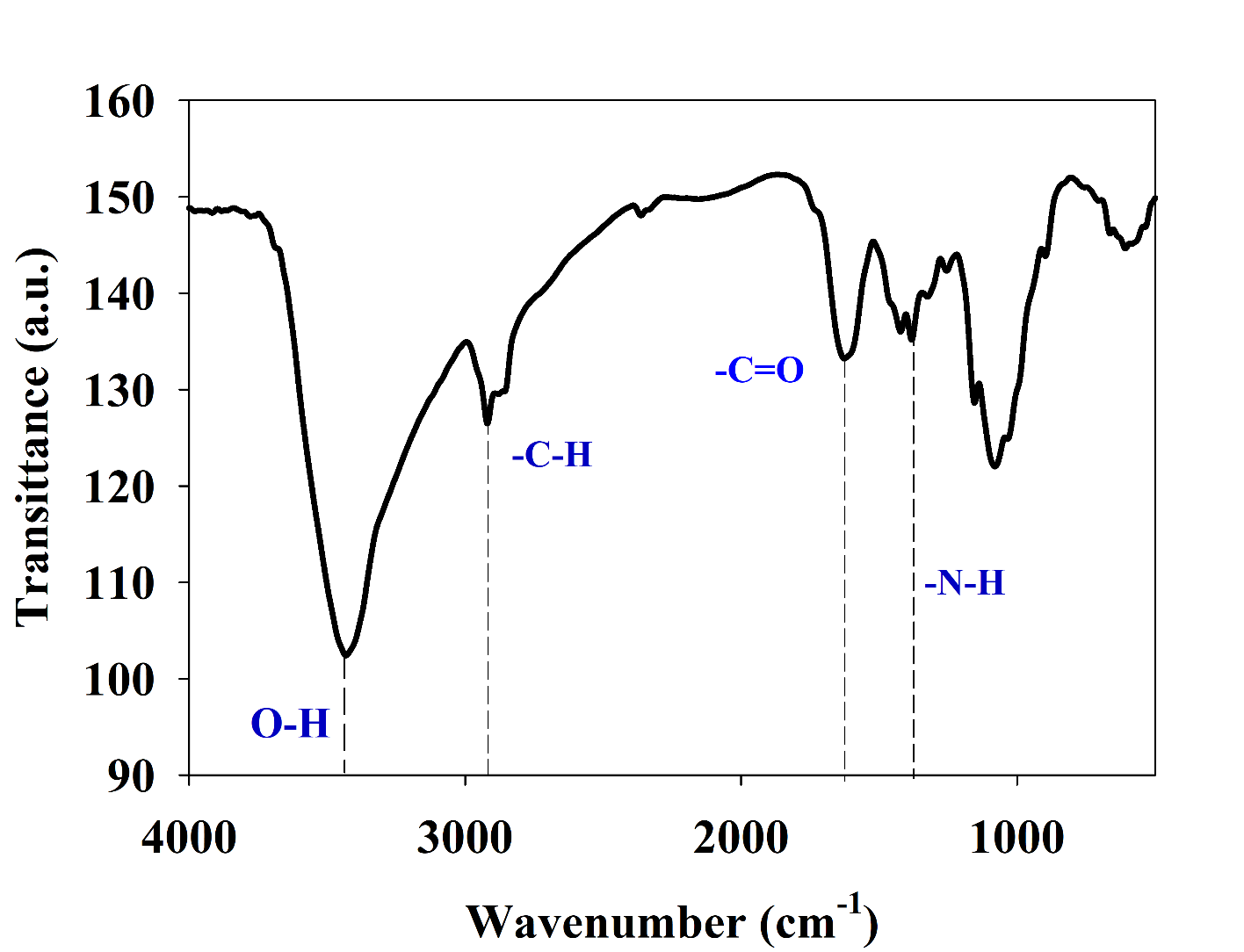


**Figure S1**. FT-IR of Chitosan sample.

**References:**

[1] M.E. Lichawska, A. Kufelnicki, M. Woźniczka, Interaction of microcrystalline chitosan with graphene oxide (GO) and magnesium ions in aqueous solution, BMC Chemistry 13 (2019) 57. https://doi.org/10.1186/s13065-019-0574-y.

[2] Y. Wang, A. Pitto-Barry, A. Habtemariam, I. Romero-Canelon, P.J. Sadler, N.P.E. Barry, Nanoparticles of chitosan conjugated to organo-ruthenium complexes, Inorganic Chemistry Frontiers 3 (2016) 1058–1064. https://doi.org/10.1039/C6QI00115G.

[3] R. Esquivel, J. Juárez, M. Almada, J. Ibarra, M.A. Valdez, Synthesis and Characterization of New Thiolated Chitosan Nanoparticles Obtained by Ionic Gelation Method, International Journal of Polymer Science 2015 (2015) 502058. https://doi.org/10.1155/2015/502058.

[4] S. Yasmeen, M.K. Kabiraz, B. Saha, M.R. Qadir, M.A. Gafur, S.M. Masum, Chromium (VI) ions removal from tannery effluent using chitosan-microcrystalline cellulose composite as adsorbent, International Research Journal of Pure and Applied Chemistry (2016) 1–14.
